# Expensive Egos: Narcissistic Males Have Higher Cortisol

**DOI:** 10.1371/journal.pone.0030858

**Published:** 2012-01-23

**Authors:** David A. Reinhard, Sara H. Konrath, William D. Lopez, Heather G. Cameron

**Affiliations:** 1 Department of Psychology, University of Virginia, Charlottesville, Virginia, United States of America; 2 Institute for Social Research, University of Michigan, Ann Arbor, Michigan, United States of America; 3 Department of Psychiatry, University of Rochester Medical Center, Rochester, New York, United States of America; 4 School of Public Health, University of Michigan, Ann Arbor, Michigan, United States of America; Semmelweis University, Hungary

## Abstract

**Background:**

Narcissism is characterized by grandiosity, low empathy, and entitlement. There has been limited research regarding the hormonal correlates of narcissism, despite the potential health implications. This study examined the role of participant narcissism and sex on basal cortisol concentrations in an undergraduate population.

**Methods and Findings:**

Participants were 106 undergraduate students (79 females, 27 males, mean age 20.1 years) from one Midwestern and one Southwestern American university. Narcissism was assessed using the Narcissistic Personality Inventory, and basal cortisol concentrations were collected from saliva samples in a laboratory setting. Regression analyses examined the effect of narcissism and sex on cortisol (log). There were no sex differences in basal cortisol, *F*(1,97) = .20, *p* = .65, and narcissism scores, *F*(1,97) = .00, *p* = .99. Stepwise linear regression models of sex and narcissism and their interaction predicting cortisol concentrations showed no main effects when including covariates, but a significant interaction, β = .27, p = .04. Narcissism was not related to cortisol in females, but significantly predicted cortisol in males. Examining the effect of *unhealthy* versus *healthy* narcissism on cortisol found that unhealthy narcissism was marginally related to cortisol in females, β = .27, p = .06, but significantly predicted higher basal cortisol in males, β = .72, p = .01, even when controlling for potential confounds. No relationship was found between sex, narcissism, or their interaction on self-reported stress.

**Conclusions:**

Our findings suggest that the HPA axis is chronically activated in males with unhealthy narcissism. This constant activation of the HPA axis may have important health implications.

## Introduction

Narcissism is a personality trait that is characterized by grandiosity, an inflated sense of self-importance, and overestimations of uniqueness [1]. For instance, narcissism is positively correlated with self references [2], self-focused attention [3], and the need for uniqueness [4]. Additionally, research has shown that non-clinical narcissists overestimate their intellectual abilities (e.g. final course grades: [5], [6]), their attractiveness [5], [7], and their positive personality traits [8].

Not surprisingly then, narcissism is associated with a number of interpersonal problems. Although people scoring high in narcissism make positive first impressions, in longer term social interactions, people interpret them more negatively [9], [10]. In romantic relationships, narcissists are low in relationship commitment, are more likely to have a ludic (game-playing) relational style, and prefer to date people who enhance their self-perceptions [11]–[13]. Narcissists score low in empathy [14] and high in hostility, with a tendency toward aggression, especially after a threat to their positive self-images [15]–[18].

Despite these negative interpersonal outcomes, narcissism is associated with a number of positive *intrapersonal* outcomes. For example, narcissists report high self-esteem [3], [4], [19]–[21], and low levels of depression, anxiety, and loneliness [22], [23]. They also tend to report more happiness and subjective well-being compared to those who score lower in narcissism [22].

Researchers have attempted to address this incongruity by positing that narcissism is multidimensional, namely, that it can be broken down into adaptive and maladaptive components. Several researchers have theorized that the most interpersonally toxic elements of narcissism are its subfactors of Exploitativeness and Entitlement [3], [4], [24]–[26], which (1) are typically uncorrelated with self-esteem, (2) are associated with increased anxiety and depression [23], [27], and (3) are the strongest predictors of poor interpersonal outcomes such as low forgiveness and increased aggression [17], [28]. Some positive intrapersonal correlates of narcissism, like high self-esteem, seem to exist *only* for the adaptive aspects of narcissism (Leadership/Authority, Superiority/Arrogance, Self-Absorption/Self-Admiration: [21], [23]). Overall, the most intrapersonally and interpersonally toxic aspects of narcissism appear to be limited to Exploitativeness and Entitlement.

### Narcissism and Defensiveness

Despite grandiose self-perceptions, many researchers find that narcissists simultaneously possess fragile self-views grounded in a sense of inferiority and worthlessness [29]. For example, Horvath and Morf [30] demonstrate that a threat to the ego activates concepts of worthlessness in those scoring high in narcissism, but has no effect on low scorers.To cope with these feelings of inferiority, narcissists use defensive strategies following threats to the self. For instance, narcissists are more likely that non-narcissists to believe that an evaluation technique is less diagnostic, and the evaluator is less competent and likeable, following negative evaluations [31]. Additionally, narcissists behave more aggressively when insulted and exhibit displaced aggression when there is a perceived threat to their perceptions of superiority [15], [32]. To bolster their sense of greatness, narcissists favor companions who enhance their self-image over caring partners [11].

### Defense Mechanisms and Physiological Reactivity

Defensive or repressive coping styles are associated with increased cardiovascular reactivity to stress, higher blood pressure, and worse outcomes of cardiovascular disease (see Rutledge, [33], for a review). These physiological responses have been confirmed using a variety of stressors and measures of defensive or repressive coping styles [34]–[39] (but see Melamed, [40], for the opposite pattern of results). Further, people with defensive coping styles do not seem to be aware of the physiological stress that their bodies are experiencing – they self-report having lower stress and increased competence when encountering stressors [34]. However, it is important to note that nearly all studies thus far have been limited to male participants [34], [36]–[40]. One study that did include female participants found that there was no interaction by sex. That is, both male and female participants who tended to use repression defensively had greater cardiovascular reactivity compared to low repressors [35].

### Narcissism and Physiological Reactivity

Given that narcissism is associated with defensive strategies, and defensiveness has physiological consequences, it would follow that narcissists may have highly reactive physiological systems. As previously mentioned, narcissists are susceptible to a host of unrealistic self-views that are difficult and stressful to continuously maintain [41]. This maintenance is likely to lead to chronic hyperactivation of the physiological stress response system, which in the long term could weaken the body's natural defenses against disease. Despite these health implications there has been limited research on physiological correlates of narcissism. We are aware of three studies that have examined cardiovascular reactivity in relation to narcissism. These studies show that narcissism is related to increased acute cardiovascular reactivity when thinking of stressful stimuli [42] or after an actual stressor [43]. Similarly, thinking of interpersonal rejection leads to an acute increase in diastolic blood pressure and heart rate for those scoring high on narcissism scales, especially on the Entitlement-Exploitativeness subscales [44].

### Narcissism and the Endocrine System

Given the cardiovascular reactivity associated with maintaining positive self-views, it stands to reason that a relationship between narcissism and hypothalamic-pituitary-adrenal reactivity would also exist. The hypothalamic-pituitary-adrenal (HPA) axis represents the key stress-response system in the body, and one marker of its activation is concentrations of salivary cortisol. During stressful events, there are acute increases of cardiovascular reactivity associated with increased cortisol (e.g. [45]). Research has found sex differences in narcissism and also in cortisol reactivity in response to stressors. Males tend to score higher on narcissism, and males also have larger acute increases in cortisol after stressors [46], [47]. So it is possible that male narcissists would be especially susceptible to increased HPA reactivity.

To our knowledge, only one study has examined the relationship between narcissism and cortisol [48]. In this study, half of the participants were randomly assigned to the Trier Social Stress Test, a laboratory task designed to elicit social-evaluative threat by requiring participants to give an impromptu speech in front of observers. The other half of the participants completed filler questionnaires for the same duration as the experimental group. Cortisol was measured at baseline (20 minutes after completing consent forms), once during the stressor (or control task) 10 minutes later, and six times more during the following 75 minutes. At baseline, there was no correlation between narcissism and cortisol (r = −0.05) in either the control group (no speech) or the experimental (speech) group. In the control group, scores on the Narcissistic Personality Inventory were not associated with changes in cortisol, and there was an overall tendency for cortisol to decline during the study period. However, after participants were told that they would have to give a speech (experimental group), narcissism was associated with a rise in cortisol and an increase in self-reported negative affect, but only for males. Narcissism scores were unrelated to cortisol or negative affect in females across both conditions.

### Current study

Research has found sex differences in narcissism and also in cortisol reactivity in response to stressors. Males tend to score higher on narcissism and have larger acute increases in cortisol after stressors [46], [47]. Further, prior work has demonstrated the role of stressful events in triggering physiological reactivity in narcissists, whether cardiovascular or endocrine. Although it is important to examine situational factors that influence the narcissism-HPA axis relationship, there is reason to predict that the HPA system may be *chronically* activated in narcissists, which could have possible implications for their health in the long-term. Specifically, chronic activation of the HPA system can lead to various problems such as suppressed immune functioning [49] and adverse cardiovascular consequences [50].

Those scoring high in Entitlement/Exploitativeness, the primary maladaptive component of narcissism, report experiencing more daily hassles or stressors, while at the same time having less available social support to deal with those hassles, compared to low scorers [51]. In addition, within normal social interactions and situations, there are a number of potentially threatening evaluations that occur every day. Although some of these might appear mundane to people low in narcissism, narcissists are highly defensive and thus may be more sensitive to such potential stressors [52]. In the single study that directly examined the relationship between narcissism and cortisol, there was no correlation between narcissism and baseline cortisol concentrations [48]. However, null results are difficult to interpret. There may be measurement differences, experimental design variations, or unknown confounds that artificially deflate a relationship that otherwise might exist. More specifically, the main focus of this prior work was on overall narcissism, but one footnote mentioned that the findings were more robust for the Entitlement/Exploitativeness subscale. This is intriguing, and in the current paper we aim to examine this possibility in depth, in the hopes of replicating and extending these findings. Thus, in the current study we examine the relationship between narcissism and cortisol under baseline (i.e. low stress) conditions, and also examine whether the more maladaptive components of narcissism are most likely to be related to increased cortisol.

## Materials and Methods

### Participants

Participants were 106 undergraduates (79 females, 27 males) recruited from a Midwestern and a Southwestern American university as volunteers or for course credit. All participants provided informed written consent prior to participating in the study. They had a mean age of 20.1 (4.2) and their ethnic composition was 70 Caucasian, 3 Asian-American, 15 Hispanic-American, 7 African-American, and 11 Other or Unidentified. All sessions were run from October to December 2009, between the weekday hours of 10 AM and 5 PM.

### Procedures

#### Salivary cortisol

At the beginning of the study, participants provided a baseline saliva sample (Time 1; 3 ml) in a sterile polypropylene tube via passive drool, completed filler tasks for approximately 25 minutes, and then provided one additional saliva sample (Time 2) in order to create a more robust basal cortisol measurement. Saliva collection is non-invasive and valid way of measuring cortisol in humans [53]. The two saliva samples had a Cronbach's alpha of 0.80 (*r* = .82, *p*<.001), and because of this, we averaged them to create a basal cortisol score for each participant. Results and conclusions remain the same regardless of whether we examine cortisol at Time 1, Time 2, or its average (see Table 1 for all results), however, for simplification we report the average cortisol results directly in the text.

**Table 1:** Regression analyses predicting cortisol from total narcissism, sex, and their interaction.

STEP 1	Average cortisol(lg)	Time 1 cortisol(lg)	Time 2 cortisol(lg)
Sex	−0.03	0.03	−0.10
Narcissism	0.23*	0.19∼	0.27*

STEP 2			
Sex	−0.04	0.02	−0.11
Narcissism	0.10	0.06	0.17
Interaction	0.23∼	0.24∼	0.18
*Females only*	0.10	0.06	0.18
*Males only*	0.42*	0.43*	0.39∼

STEP 3			
Sex	−0.01	0.03	−0.06
Narcissism	0.05	0.00	0.15
Interaction	0.27*	0.28*	0.21∼
Positive Mood	0.02	0.11	−0.13
Negative Mood	0.10	0.11	0.10
Stressed (*1 = not at all, 5 = completely*)	−0.05	−0.06	−0.04
Social Support (1 = yes, 0 = no)	0.05	0.05	0.06
Relationship status (*1 = committed relationship, 0 = not in committed relationship*)	−0.24*	−0.24*	−0.20∼

∼ p<.10,

*p<.05,

**p<.01.

Saliva samples were sealed and frozen at −20 Celsius until they were assayed at the Core Assay Facility in the University of Michigan Psychology Department. Enzyme immunoassay (EIA; Salimetrics) was used to ascertain participant cortisol concentrations. Cortisol was measured using 1∶20 water-diluted standards combined with 150 µl cortisol samples (cortisol range: 0.019–1.079 µg/dL). Average lower limits of detection (Mean B0 - 2*SD) were calculated on 10 sets of duplicates at the 0 µg/dL level, leaving a minimal cortisol concentration of <0.003 µg/dL. The manufacturer provided analytic recovery values for diluted lyphochek control samples for four dilution factors ranging from 1∶2 to 1∶16, with corresponding analytic recovery values ranging from 80.1% to 97.9%. Samples were analyzed in six assays, with inter-assay Coefficients of Variability (CV) ranging from a low value of 7.5% to a high value of 12.4%, and intra-assay CVs ranging from a low value of 3.7% and a high value of 7.1% Inter-assay %CVs less than 15 and intra-assay %CVs less than 10 are considered acceptable by the EIA manufacturer (Salimetrics). Samples were assayed for cortisol, and one outlier was removed before completing the analyses (over 8 SDs above mean).

The relationship between time of participation (coded using a 24 hour clock; from 10:00 to 17:00) and participant average salivary cortisol concentrations was negative, but non-significant (Time 1: *β* = −0.14, *p* = .15; Time 2: *β*  = −0.01, *p* = .94; Average cortisol: *β* = −0.10, *p* = .32). Although this lack of significance is surprising given diurnal rhythms in cortisol concentrations (e.g. [54], [55]), there are a number of individual differences that affect the steepness of the decline in cortisol throughout the day (e.g. age, depression, social support; 54). Moreover, in a college student sample it is possible that participants arriving to a study in the early afternoon would still be experiencing their cortisol awakening response (which peaks 30 minutes after waking up) if they had slept in, which would artificially reduce the steepness of the cortisol decline. Future studies should assess time of awakening in addition to time of participation.

Although we did not collect data regarding hormonal contraception use among our participants, given the mixed data regarding its impact on cortisol concentrations, it is unclear to what extent this could explain our null results among women. While some research has shown that women taking hormonal contraceptives have increased production of corticosteroid binding globulin (CBG) compared to women not taking hormonal contraceptives [56]–[58], the impact of hormonal contraceptives on free salivary cortisol concentrations is less clear. Several studies have shown no differences in baseline cortisol concentrations among women using hormonal contraception compared to those not using it [57], [59], [60], while others have shown higher baseline cortisol concentrations in contraceptive users [58], [61].

#### Questionnaire measures

Narcissism was measured using the 40-item Narcissistic Personality Inventory (NPI-40; [62]). For each of the forced-choice dyads on the scale, participants chose either the narcissistic response (e.g., “*If I ruled the world it would be a better place*”) or the non-narcissistic response (e.g., “*The thought of ruling the world frightens the hell out of me*”). The total number of narcissistic responses were summed together, with higher scores indicating higher levels of narcissism. We also examined whether specific narcissism subfactors were related to cortisol. An *unhealthy narcissism* score was created by summing the Entitlement and Exploitativeness subscales of the NPI. A *healthy narcissism* score was created by summing the Leadership/Authority, Self-Sufficiency, Superiority, and Vanity subscales of the NPI. Prior research has validated this conceptualization of narcissism [23].

Mood was measured with the Positive and Negative Affect Schedule (PANAS) [63], in which participants are asked to what extent they were currently feeling 10 positive and 10 negative affective terms (1 = *very slightly or not at all*, 5 = *extremely*). In addition, we also assessed participants' general stress levels by asking them the following question: “In general, how stressed out have you been within the past week?” (*1 = not at all, 5 = completely*). Finally, we asked participants whether they believed that they were getting the social and emotional support that they need (*1 = yes, 0 = no*) and to report their relationship status (*1 = committed relationship, 0 = not in committed relationship*).

## Results

### Descriptive statistics

The average cortisol concentration of participants was .21 µg/dL (*SD* = .18) and the average narcissism score was 17.88 (6.59). There were no sex differences in basal cortisol: males (*M* = .23, *SD* = .23) had similar concentrations of cortisol as females (*M* = .20, *SD* = .16), *F*(1,103) = .92, *p* = .23. In addition, males (*M* = 17.89, *SD* = 7.20) and females (*M* = 17.88, *SD* = 6.42) had nearly identical narcissism scores, *F*(1,97) = .00, *p* = .99. There were no relationships between cortisol and self-reported stress, *r*(95) = .06, *p* = .59, social support, *r*(96) = .02, *p* = .87, or relationship status, *r*(86) = −.13, *p* = .23. There were also no relationships between narcissism and stress, *r*(96) = −.04, *p* = .70, social support, *r*(97) = .03, *p* = .75, and relationship status, *r*(86) = −.08, *p* = .45.

### Overall narcissism and cortisol

**Figure 1:**
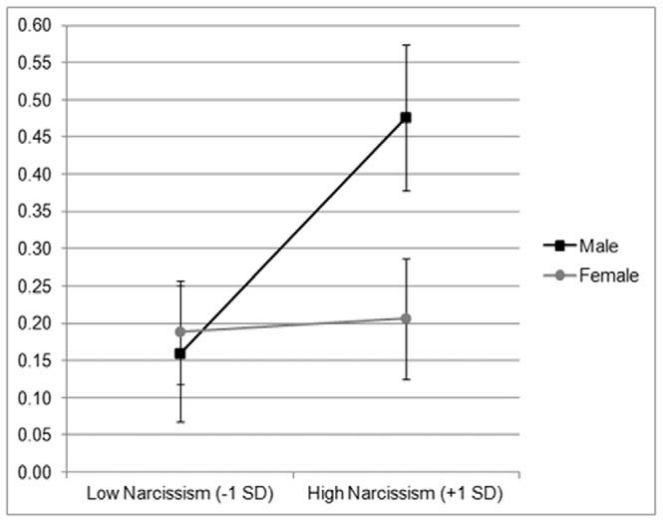
Raw cortisol concentrations of men and women who were low (−1 SD) and high (+1 SD) in narcissism (standard errors in parentheses).

**Figure 2:**
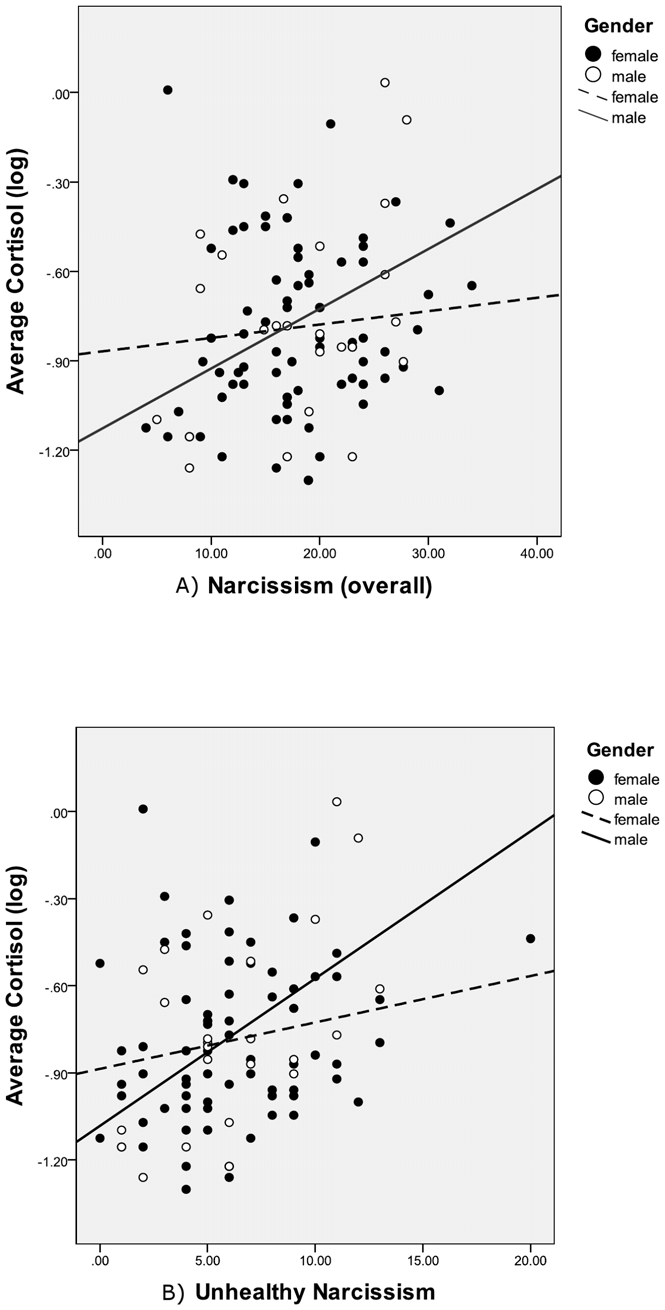
Scatterplot depicting the relationship between cortisol concentrations (log) and (a) overall narcissism, and (b) unhealthy narcissism, with overlaid regression lines.

For the purpose of data analysis, cortisol concentrations were log-transformed to reduce skewness. Raw values in µg/dL are presented in Figure 1 and logged values are presented in Figure 2. A stepwise linear regression was used to examine the effects of sex and narcissism on the log of cortisol concentrations. We entered sex and (mean-centered) narcissism into the regression model in Step 1, followed by their interaction in Step 2, and covariates (mood, general stress, social support, and relationship status) in Step 3.

In Step 1, there was no main effect of sex, *β* = −.03, *p* = .80, but there was a main effect of narcissism on the log of basal cortisol, *β* = .23, *p* = .039. (The adjusted R^2^ for Step 1 was 2.8%.) In Step 2, there was no main effect of sex, *β* = −.04, *p* = .71, or narcissism, *β* = .10, *p* = .45, on the log of basal cortisol, but their interaction was marginally significant, *β* = .23, *p* = .08. (The adjusted R^2^ for Step 2 was 5.3%.) In Step 3, this interaction became significant when controlling for mood, general stress, social support, and relationship status, *β* = .27, *p* = .04. (The adjusted R^2^ for Step 2 was 6.1%.) For simplicity, we focus on average cortisol results, but Table 1 presents results from each separate cortisol assessment (Time 1 and Time 2) as well as average cortisol concentrations.

To investigate the interaction between sex and narcissism, we split the sample by sex and regressed narcissism on the log of cortisol concentration (See Figure 1 for visual depiction at +/−1 SD of narcissism). We found that narcissism was unrelated to the log of cortisol in females, *β* = .10, *p* = .39 (Adj. R^2^ = −0.30%), but significantly predicted the log of cortisol in males, *β* = .42, *p* = .038 (Adj. R^2^ = 13.9%).

### Type of narcissism and cortisol

We next conducted a stepwise linear regression with sex, *unhealthy* narcissism (centered), and *healthy* narcissism (centered) predicting the log of average basal cortisol in Step 1, the interaction between sex and both types of narcissism predicting the log of cortisol in Step 2, and covariates (mood, general stress, social support, and relationship status) in Step 3. (Step 1 explained 8.1% of the variance in basal cortisol, Step 3 explained 12.1% of it, and Step 3 explained 12.9% of it.)

We were specifically interested in whether the most toxic aspects of narcissism were associated with higher cortisol in males. In Step 1, there was no effect of sex, *β* = −.04, *p* = .73, or healthy narcissism on the log of cortisol, *β* = −.12, *p* = .36. However, participants with higher unhealthy narcissism also had higher cortisol, *β* = .39, *p* = .003. In Step 2, there were no effects of sex, *β* = .39, *p* = .30, or healthy narcissism, *β* = −.16, *p* = .27, and no interaction between sex and healthy narcissism, *β* = −.05, *p* = .84. However, there was a significant main effect of unhealthy narcissism on the log of cortisol, *β* = .27, *p* = .047, and a nearly significant interaction between sex and unhealthy narcissism, *β* = .49, *p* = .064. In Step 3, this interaction became significant when controlling for mood, general stress, social support, and relationship status, *β* = .51, *p* = .050 (See Table 2).

**Table 2:** Regression analyses predicting cortisol from healthy and unhealthy narcissism, sex, and their interaction.

STEP 1	Average cortisol(lg)	Time 1 cortisol(lg)	Time 2 cortisol(lg)
Sex	−0.03	0.02	−0.11
Healthy narcissism	−0.12	−0.12	−0.08
Unhealthy narcissism	0.39**	0.36**	0.39**

**STEP 2**			
Sex	0.39	0.38	0.36
Healthy narcissism	−0.16	−0.17	−0.09
Unhealthy narcissism	0.27*	0.25∼	0.29*
Sex X healthy narcissism	−0.05	−0.01	−0.13
Sex X unhealthy narcissism	0.49∼	0.45∼	0.48∼
*Females only: healthy narcissism*	−0.13	−0.13	−0.08
*Females only: unhealthy narcissism*	0.27∼	0.22∼	0.30*
*Males only: healthy narcissism*	−0.30	−0.29	−0.28
*Males only: unhealthy narcissism*	0.72*	0.71*	0.67*

**STEP 3**			
Sex	0.41	0.38	0.40
Healthy narcissism	−0.18	−0.22	−0.09
Unhealthy narcissism	0.25∼	0.23∼	0.26∼
Sex X healthy narcissism	−0.01	0.04	−0.09
Sex X unhealthy narcissism	0.51*	0.48∼	0.50∼
Positive Mood	0.05	0.14	−0.09
Negative Mood	0.08	0.09	0.08
Stressed (*1 = not at all, 5 = completely*)	−0.03	−0.04	−0.01
Social Support (1 = yes, 0 = no)	0.11	0.11	0.10
Relationship status (*1 = committed relationship, 0 = not in committed relationship*)	−0.22*	−0.23*	−0.19∼

∼ p<.10,

*p<.05,

**p<.01.

When splitting by sex, we found that *healthy* narcissism was not related to the log of cortisol in either males, *β* = −.30, *p* = .26, or females, *β* = −.13, *p* = .36. Unhealthy narcissism, on the other hand, was associated with marginally higher cortisol in females, *β* = .27, *p* = .055, and significantly higher cortisol in males, *β* = .72, *p* = .011. In fact, unhealthy narcissism was more than twice as large a predictor of cortisol in males as in females.

### Narcissism and stress

We next ran a stepwise linear regression to examine the effects of sex, narcissism, and their interaction, on self-reported stress. No effects emerged as significant, either with narcissism overall or with the healthy versus unhealthy subscales, *ps*>.55.

## Discussion

The present study examined the relationship between narcissism and basal cortisol concentrations in male versus female participants. Previous experiments have studied the role of narcissism and reactivity to acute stressful events, and we sought to determine whether narcissists have higher basal cortisol concentrations even without an explicit experimentally induced stressor. Participants gave two saliva samples (one directly after consent and the second after 25 minutes) to determine a baseline concentration of salivary cortisol. We found that narcissism predicted higher basal cortisol concentrations overall, and especially in males, even when controlling for mood, general stress, social support, and relationship status.

A novel aspect to the current study was our analysis of healthy versus unhealthy types of narcissism. We found that higher *unhealthy* narcissism predicted higher salivary cortisol concentrations in males but there was no relationship between *healthy* narcissism and cortisol in males. This pattern remains when adding important controls. This is consistent with prior work showing that unhealthy narcissism might be the most important aspect of narcissism to examine with respect to cortisol [48]. In females, there is a marginal tendency for women with high *unhealthy* narcissism to have higher cortisol concentrations, however, the effect size is over 2.5 times smaller in females (*β* = .27) compared to males (*β* = .72). Overall, the relationship between narcissism and cortisol in females is less clear, and warrants further research.

These findings extend previous research by showing that narcissism may not only influence how people respond to stressful events, but may also affect how they respond to their regular day-to-day routines and interactions. In a recent study examining the relationship between narcissism and cortisol, it was found that higher narcissism predicted greater cortisol reactivity to a laboratory-induced stressful event in males [48]. However, these authors did not find a relationship between *basal* cortisol and narcissism in males. Between subtle contextual factors and confounding variables, it is difficult to explain why this particular null effect exists. Yet, one important difference between the current study and Edelstein et al [48] was the operationalization of baseline cortisol concentrations. Edelstein and colleagues recorded basal saliva measures 20 minutes after an adaptation (i.e. relaxation) period whereas our basal saliva was taken at two time points: directly after consent and after 25 minutes of filler tasks. There are advantages and disadvantages to both methods. One benefit to Edelstein and colleagues' method is it allows the person to acclimate and adjust to a new environment before giving a sample. However, one advantage to measuring a baseline without a relaxation period is that it captures a more realistic account of how people typically respond to everyday situations. We found that regardless of whether we examined the Time 1, Time 2, or average cortisol concentrations, our results were similar (See Tables 1 and 2).

Males tend to score higher on narcissism, and males also have larger increases in cortisol concentrations after stressors [46], [47]. Our findings suggest that the HPA axis may be chronically activated in males high in unhealthy narcissism, even without an explicit stressor. Given societal definitions of masculinity that overlap with narcissism (i.e. they include arrogance and dominance), we hypothesize that these difficulties in maintaining an inflated sense of the self are at least in part related to the extent to which males endorse stereotypically male gender roles. Threats to male gender roles and masculinity are constant, and provide a source of stress that make these roles difficult to maintain [64], [65]. Narcissism is also stressful and difficult to maintain [41]. In addition, both high masculinity and narcissism advocate for high independence and agency, and emphasize individualism over an acceptance of social support [51], [66]. Because high narcissists report experiencing a greater number of daily hassles compared to low narcissists, low social support is especially likely to be toxic. It is important to note, however, that we know that lack of social support itself is not the only explanation for our findings, as relationships between narcissism and cortisol remain even after statistically controlling for perceived social support.

Why do unhealthy aspects of narcissism influence males and females differently? Perhaps females can escape more severe physiological consequences of narcissism because there are different expectations for their roles in society. Female gender roles promote behaviors that encourage women to value relationships and to seek and gain social support [67]–[69], which may lower their risks for chronic activation of the HPA axis. In fact, female narcissism might be associated with different kinds of exploitative strategies than male narcissism. Perhaps female narcissists use “feminine” roles to their advantage and obtain both social and financial resources more indirectly. This is an untested hypothesis so far, but may be worth exploring in future research in order to understand why narcissism does not appear to be as physiologically taxing for women as it is for men.

One of the limitations of our study is its correlational nature. Accordingly, the direction of causality is unclear, and there is also a possibility of unknown confounds influencing the results (e.g. testosterone levels among participants, which were not assessed). However, we found that our effect is still robust while controlling for other plausible explanatory variables such as mood, general stress, social support, and relationship status. Finally, our sample is relatively small and taken from a relatively homogenous population (i.e. college students), with a higher proportion of females relative to males (79 females to 27 males). However, given the significant findings among the relatively small male population, we would expect our results to be even more robust in a larger sample. In any case, future research would need to replicate these findings in larger and more representative samples.

Given our preliminary findings of higher cortisol among unhealthy narcissists, future investigations should examine potential links with other physiological responses also related to cortisol and stress. Corticotropin releasing hormone (CRH) is a stress hormone released from the hypothalamus that triggers a series of biological reactions leading to the production of cortisol [70]. Unfortunately, collecting biological samples to measure CRH has been proven to be difficult, as concentrations are very small in non-pregnant women, and are typically collected via blood serum [71]. However, other biological measures implicated in increased psychological stress and poor coping include inflammatory markers such as interleukin-6 (Il-6) and C-Reactive Protein (CRP) [72], making both of these promising future directions for exploring other physiological correlates of extreme self-focus.

In conclusion, our findings highlight the possibility that for males, narcissism may have an especially negative physiological effect. Considering the rising narcissism among both men and women in American culture [73], there may be potential long-term public health consequences if these trends continue. Given research finding that chronic HPA activation is associated with cardiovascular problems [50], and other work finding that an increased use of first-person singular pronouns is also associated with poor cardiovascular health [74], [75], future work might examine high narcissism in earlier life predicts poor health outcomes in later life. We also recommend that future research attempt to better understand *why* male narcissists have higher basal cortisol concentrations, and in doing so, help to pinpoint potential windows of intervention.
