# Correction: Expensive Egos: Narcissistic Males Have Higher Cortisol

**DOI:** 10.1371/annotation/1c60eca3-794f-4a09-8a82-e43ed3cc2009

**Published:** 2012-07-12

**Authors:** David A. Reinhard, Sara H. Konrath, William D. Lopez, Heather G. Cameron

This article was made available as Open Access with the support of the University of Michigan COPE Fund, http://lib.umich.edu/cope

